# Correlation of TTLL7-IT1/Hsa-miR-29c-3p/GLS with limited cutaneous systemic sclerosis and exploration of the underlying mechanisms

**DOI:** 10.3389/fimmu.2026.1722661

**Published:** 2026-05-21

**Authors:** Xin Dai, Shanyu Chen, Danni Zhu, Guoxing Ma, Bo Liang, Bing Chen, Yan Liu, Xue Liu, Jiejie Qiao, Jihong Hu, Yijing Zhao, Jiaye Fei, Xiyuan Bao, Kangrui Xia, Haifeng Pan, Shengquan Zhang, Jing Wang

**Affiliations:** 1 Department of Epidemiology and Biostatistics, School of Public Health, Anhui Medical University, Center for Big Data and Population Health of Institute Health and Medicine, Institute Health and Medicine, HeFei Comprehensive National Science Center (IHM), The First Affiliated Hospital of Anhui Medical University, Rheumatology and Immunology Department, Clinical College of Anhui Medical University, Key Laboratory of Public Health Social Governance, Philosophy and Social Sciences of Anhui Province, Hefei, Anhui, China; 2 The First Affiliated Hospital of Anhui Medical University, Rheumatology and Immunology Department, Hefei, Anhui, China; 3 Department of Chronic Non-Communicable Diseases Prevention and Control Fei Xi County Centre for Disease Control and Prevention, Hefei, Anhui, China; 4 The First Affiliated Hospital of Anhui Medical University, Institute of Dermatology and Department of Dermatology, Key Laboratory of Dermatology, Anhui Medical University, Ministry of Education, Hefei, Anhui, China; 5 Department of Biochemistry and Molecular Biology, School of Basic Medical Sciences, Anhui Medical University, Hefei, Anhui, China; 6 Department of Public Health and Health Management, Clinical College of Anhui Medical University, Hefei, Anhui, China; 7 School of Public Health, Anhui Medical University, Hefei, Anhui, China; 8 Clinical Medicine in the 5 + 3 Integrated Program, Anhui Medical University, Hefei, Anhui, China; 9 Department of Epidemiology and Biostatistics, School of Public Health, Anhui Medical University, Hefei, Anhui, China

**Keywords:** CD8+ T cell, ceRNA network, cuproptosis, *Glutaminase*, limited cutaneous systemic sclerosis

## Abstract

**Background:**

Systemic sclerosis (SSc) is an autoimmune disease. In the types of SSc, lcSSc presents with milder symptoms and poses challenges for early diagnosis. Therefore, the identification of biomarkers for earlier diagnosis and the discovery of potential therapeutic targets are of greater significance. This study aims to explore the relationship between competing endogenous RNA (ceRNA) networks, cuproptosis, and lcSSc.

**Methods:**

RNA sequencing was performed to profile differentially expressed RNAs in blood samples from lcSSc patients and healthy controls. The potential ceRNA network targeting relationships were then examined using a dual-luciferase reporter assay. Subsequently, a series of *in vitro* experiments utilizing primary CD8^+^ T cells. These experiments, which included phenotypic assays and molecular analyses, demonstrated the occurrence of cuproptosis. Finally, these findings were validated *in vivo* in a mouse model through analyses of skin tissues using H&E staining, Masson staining, Immunofluorescence, qPCR, and Western blotting.

**Results:**

TTLL7-IT1/hsa-miR-29c-3p/GLS was constructed using bioinformatic analysis of lcSSc. CD8^+^ T cells from patients with lcSSc exhibited elevated copper ion levels and reduced glutaminase(GLS) expression, accompanied by alterations in their phenotype and cuproptosis-related proteins. These phenotypic and protein expression changes were reversed by cuproptosis inhibition. Furthermore, cuproptosis exacerbates fibrosis and vascular pathology. Animal experiments further revealed that in bleomycin-induced mouse models, both inhibition of copper ions and GLS overexpression reduced dermal thickness, collagen deposition, fibrotic factor expression, and CD31/α-SMA co-localization in the skin.

**Conclusions:**

In summary, our findings reveal a functional interaction between cuproptosis and ceRNA networks: TTLL7-IT1/hsa-miR-29c-3p/GLS influences cuproptosis and lcSSc progression, providing new insights into the pathogenesis of lcSSc.

## 1 Introduction

Systemic sclerosis (SSc) is a chronic autoimmune disease with a high prevalence in women. It has a globally estimated incidence of approximately 1.4–8.6 per 100,000 person-years, and the pooled prevalence is estimated to be between 17.6 and 18.9 per 100,000 person-years, with notable regional variations (1, 2). SSc is classified into limited cutaneous SSc (lcSSc) and diffuse cutaneous SSc(dcSSc). Furthermore, lcSSc is more prevalent than dcSSc, but it is associated with fewer skin lesions and milder symptoms and the complexity and heterogeneity of SSc pathogenesis contribute to this wide spectrum of disease manifestations (3, 4). Consequently, diagnosis was often delayed, making early clinical detection more difficult. Therefore, it is crucial to deepen our understanding of the pathogenesis of lcSSc for the identification of early diagnostic biomarkers and therapeutic targets, which will facilitate the early detection, diagnosis, and treatment of affected patients.

When studying the mechanism of lcSSc, researchers have increasingly directed their attention toward long noncoding RNAs (lncRNAs) as promising avenues for elucidating its molecular pathogenesis. LncRNAs can bind to microRNAs (miRNAs), ultimately mediating the formation of competing endogenous RNA (ceRNA) networks derived from mRNAs, which are likely to influence physiological processes in various diseases. ceRNA networks play important roles in rheumatoid arthritis, ankylosing spondylitis, and systemic lupus erythematosus (5–9). However, only a few reports exist on ceRNA networks in SSc, moreover, existing ceRNA network research have not differentiated between dcSSc and lcSSc (10, 11). Although some miRNAs (12), such as miR-143-3p, have been identified to improve fibrosis in lcSSc (13), the role of ceRNA in lcSSc remains incomplete and requires further research.

A new cell death mechanism distinct from ferroptosis, called cuproptosis, has been reported in autoimmune diseases (14, 15). In 2022, Tsvetkov proposed that Cu ions are essential for maintaining cellular homeostasis; however, they become cytotoxic when their concentrations exceed the homeostatic threshold. Excessive reactive oxygen species (ROS) are produced during this process (16). Although a direct connection between cuproptosis and lcSSc has not yet been reported, the existing literature provides some evidence. Elevated serum copper levels have been observed in patients with SSc (17). In addition, exposure to elevated copper ion levels in the respiratory tract lining fluid contributes to oxidative stress in patients with SSc (18). ROS is a key driver of SSc, modulating key pathways and propelling disease advancement, demonstrating that alterations in copper levels may be responsible for the elevated inflammation and oxidative stress observed in individuals with lcSSc through ROS production. The primary source of ROS in humans is the tricarboxylic acid (TCA) cycle, which occurs in the mitochondria during cuproptosis. During cuproptosis, intracellular copper ion levels increase, leading to increased ROS production via the TCA cycle (19, 20).

The effect of cutaneous cuproptosis on the clinical manifestations of lcSSc can be understood by studying cancers with characteristics similar to those of SSc. Studies in oncology suggest that cuproptosis may modulate immune activity via CD8^+^ T cells. In microsatellite-stable colorectal cancer, cuproptosis enhances the cytolytic function of CD8^+^ T cells and tumor cell oxidative stress while activating pathways related to immunity and inflammation (21). In addition, islet amyloid polypeptide inhibits CD8^+^ T cell antitumor activity by targeting apoptosis (22). CD8^+^ T cells are maintained within a stable range in the body, and deviations from this equilibrium can cause immune disorders and deficiencies (23). Their aberrant activation drives autoimmune responses and inflammation. These studies suggest a latent role for cuproptosis in the pathogenesis of lcSSc. Researchers have constructed ceRNA networks related to cuproptosis in atherosclerosis, a chronic inflammatory disease in which these networks modulate mitochondrial function and disease progression (24). Endothelial damage is an early pathological characteristic of atherosclerosis, and late-stage atherosclerotic plaques exhibit fibrotic characteristics similar to those of lcSSc.

Therefore, based on the evidence, this study aimed to investigate the potential mechanisms by which the ceRNA network may contribute to lcSSc through cuproptosis, regulation of CD8^+^ T cells, and other pathways. Our study provides new perspectives and directions for early intervention and targeted therapy in patients with lcSSc.

## 2 Materials and methods

### 2.1 Samples

#### 2.1.1 Cases and controls

Study participants were recruited from individuals diagnosed with lcSSc at the Rheumatology Department of the First Affiliated Hospital of Anhui Medical University. Adherence to the diagnostic and classification standards for SSc, set forth by the American College of Rheumatology and European League Against Rheumatism diagnostic criteria in 2013 (25), was mandatory for inclusion in the study. lcSSc was diagnosed by immuno-rheumatologists. For some basic information on lcSSc and HC, please refer to **Table 1**.

**Table 1:** Demographic information for lcSSc patients and controls.

Characteristics	Patients with lcSSc	Healthy control
Number	20	20
Gender (M/F)	M (4)/F (16)	M (4)/F (16)
Height	160.4 ± 5.698	160 ± 5.859
Age (mean ± SD)	65 ± 8	64.7 ± 8.5
Weight(kg)	55.75 ± 12.965	57.1 ± 12.546

Healthy controls (HC) samples were collected from the Department of Physical Examination, whose blood counts, urinary counts, and liver and renal function tests were within the normal range. Controls were matched with patients according to age and sex. None of the HC had autoimmune or neoplastic diseases or any other serious illnesses. For some basic information on lcSSc and HC, please refer to **Table 1**.

#### 2.1.2 The modified Rodnan Skin Score

A trained professional performs the modified Rodnan Skin Score on the patient’s left and right fingers, left and right hands, left and right forearms, left and right upper arms, face, anterior chest, abdomen, left and right thighs, left and right calves, and left and right feet to assess the degree of skin thickening. The scores are then summed to obtain the total score. The scoring criteria were as follows: 0 = normal skin; 1 = mild thickening and hardening; 2 = moderate hardening (cannot be lifted); 3 = severe hardening (cannot be moved) (26).

#### 2.1.3 Mice

C57BL/6 mice (6–8 weeks old) weighing approximately 20 g were purchased from Spefu Biotechnology Co., Ltd. (Beijing, China). The housing conditions for the mice were as follows: temperature, 22 °C; humidity, 50–60%; fresh indoor air; disinfected cages; disinfected purified water; and rat feed. Thirty mice were randomly assigned to five groups: healthy mice (injected with equal amounts of saline), lcSSc model (injected with Bleomycin (BLM)), lcSSc+TTM (injected with BLM+TTM), GLS-overexpression (injected with BLM overexpressing GLS virus), and control (injected with BLM overexpressing NC virus). Subcutaneous injection of BLM constructs in SSc model mice is commonly used as a mice model for lcSSc, as the pathological changes in the dermis resemble those observed in lcSSc patients (13). BLM was administered via subcutaneous injection into the backs of mice for 28 consecutive days (dissolved in saline at a dose of 1 mg/kg/day based on body weight). TTM (0.5 mg/kg/day, dissolved in a solvent selected according to the manufacturer’s instructions) was administered daily starting on day 14 of model induction, into the area surrounding the BLM injection site, and continued until the final day of BLM administration. For GLS overexpression, AAV-GLS (2×10^13^ vg/mL) was injected on day 14 of model induction; 3 μL was administered at each of five perilesional points surrounding the BLM injection site (total volume: 15 μL per mouse). Skin tissue was harvested 24h after the final BLM injection for subsequent experiments.

Mice were anesthetized with a 1.5%–2.5% isoflurane-oxygen mixture for 5–10 minutes and then euthanized by cervical dislocation. BLM (Zhejiang Hai Zheng Pharmaceutical Co., Ltd., Taizhou, China), TTM (Merck, 323446) and isoflurane (Shandong Ante Animal Husbandry Technology Co., Ltd., R510-22-16) were used in this study.

### 2.2 Methods of cuproptosis-related ceRNA network construction and identification of immune cells

#### 2.2.1 Bioinformatics analysis

We performed 5-to-5 transcriptome sequencing of PBMC and used the clusterProfiler R language package to analyze the differentially expressed genes in terms of Gene Ontology (GO) and Kyoto Encyclopedia of Genes and Genomes (KEGG) pathway enrichment. To predict the upstream and downstream regulators of miRNAs, the obtained miRNA-mRNA interaction pairs (derived from sequencing data) were cross-referenced with RNA interaction databases, including starBase v2.0 and BiBiServ.

#### 2.2.2 Immune infiltration

Transcriptome sequencing data of lcSSc PBMC were analyzed using CIBERSORTx for 22 immune cell types from PBMC samples.

### 2.3 Cell experiments

#### 2.3.1 Cell culture and process

All CD8^+^ T cell extraction, processing, and detection procedures undertaken during this experiment were performed by immediately transferring patient blood samples, collected using EDTA blood collection tubes, to the laboratory for fresh extraction. Primary CD8^+^ T cells were extracted using a cell selection kit (IPHASE, 071A403.14). activated with CD3/28 (Stemcell,10971) and cultured in ImmunoCult-XF T Cell Exp Medium (Stem Cell, 10981) or 1640 (Gibco,11875093) supplemented with IL-2 (MCE, HY-P7037). A growth medium comprising DMEM (Gibco, 6124526), 10% fetal bovine serum (FBS, WISENT, 086-150), and 1% penicillin-streptomycin (P/S, Beyotime, C0222) was used to maintain 293T cells (Feng Hui Biotechnology, China). Human skin fibroblasts (HSF) and human umbilical vein endothelial cells (HUVEC) (Feng Hui Biotechnology, China) were grown in a medium supplemented with specific growth factors. Jurkat cells were cultured in RPMI 1640 medium supplemented with 10% FBS and 1% P/S. All cells were cultured at 37 °C in 5% CO_2_. To inhibit apoptosis, CD8^+^ T cells were treated with ammonium tetrathiomolybdate (TTM; Merck, 323446).

#### 2.3.2 Flow cytometry

Detection of the relative content of CD8^+^ T cells: Fresh blood was collected, and antibodies (Anti-Human CD45, APC, Lianke Bio, 70-F1104503-25; PE anti-Human CD3, Biolegend, 317307; Anti-Human CD8α, APC-Cy7, Lianke Bio, F11008A06) were added. After centrifugation, cleaning, resuspension, and filtration, the mixture was analyzed by flow cytometry. FlowJo 10.8.1 was used to analyze and generate flow cytometry images.

ROS and CD8^+^ T cells were collected after incubation. Following the instructions of the ROS Detection Kit, the cells were reconstituted in serum-free medium (Beyotime, China) and analyzed using a flow cytometer. FlowJo 10.8.1 was used to analyze and generate flow cytometry images.

#### 2.3.3 RT-qPCR

Following the standard protocol, RNA was extracted from the PBMC samples using TRIzol reagent (Invitrogen, Carlsbad, CA, United States). LncRNAs and mRNA were reverse transcribed into complementary DNA (cDNA) using an RT kit (Takara RR047A). miRNA reverse transcription was performed using a stem-loop method kit (GenStar, A237-10), qPCR was performed according to the instructions (Takara, RR820A). Relative expression of candidate RNAs was normalized to the housekeeping genes GAPDH/β-actin (lncRNA and mRNA) or U6 (miRNA) and calculated using the 2^-ΔΔCt^ method. To ensure accuracy and precision, all samples were subjected to triplicate measurements in the quantitative assays, and the results were expressed as the mean of the replicates. *Glutaminase(GLS), TTLL7-IT1, Hsa-miR-29c-3p, CD31, COL1A1, α-SMA*, and *FN1* were detected. The primer sequences used were presented in **Table 2**.

**Table 2:** Primer sequences (humans and mouse).

Species	Gene	Primer	Sequence (5′→3′)
Human	GLS	Forward	ATTCAGTCCCGATTTGTGGGG
		Reverse	AGAAGGGAACTTTGGTATCTCCA
Human	TTLL7-IT1	Forward	TGCCATAAGCAAGTCCTGCAA
		Reverse	CTAAGTACTCTTGGGGTAATCCCG
Human	CD31	Forward	AACAGTGTTGACATGAAGAGCC
		Reverse	TGTAAAACAGCACGTCATCCTT
Human	COL1A1	Forward	AAGCGTGAGTCGCAAGAATG
		Reverse	TCTCCAGGTTTTCGCCAGTG
Human	α-SMA	Forward	GACAATGGCTCTGGGCTCTGTAA
		Reverse	TGTGCTTCGTCACCCACGTA
Human	Fibronectin 1	Forward	AGGAAGCCGAGGTTTTAACTG
		Reverse	AGGACGCTCATAAGTGTCACC
Human	GAPDH	Forward	GGAGTCCACTGGCGTCTTCA
		Reverse	GCAGAGGGGGCAGAGATGAT
Human	U6	Forward	GTGCTCGCTTCGGCAGCACATA
		Reverse	GCGCAGGGGCCATGCTAATCTTC
Human	Hsa-miR-29c-3p	RT	GTCCTCCTCTCCTCTCCTCTCATGAGGAGGACTAACCG
Human	Hsa-miR-29c-3p	Forward	AGGGCTAGCACCATTTGAAAT
		Reverse	TCCTCCTCTCCTCTCCTCTC
Mouse	Mo-β-actin	Forward	AGTGTGACGTTGACATCCGT
		Reverse	TGCTAGGAGCCAGAGCAGTA
Mouse	Mo-GAPDH	Forward	GGGTCCCAGCTTAGGTTCAT
		Reverse	CCAATACGGCCAAATCCGTT
Mouse	Mo-α-SMA	Forward	GTCCCAGACATCAGGGAGTAA
		Reverse	TCGGATACTTCAGCGTCAGGA
Mouse	Mo- Fibronectin 1	Forward	AAATCGTGCAGCCTCAATCC
		Reverse	GGCTTGCTCTCGCAGTTAAA
Mouse	Mo-COL1A1	Forward	AACTGGTACATCAGCCCGAA
		Reverse	TTCCGTACTCGAACGGGAAT
Mouse	Mo-GLS	Forward	AAACCTTCTGTTTGCCGCAT
		Reverse	TCATAATCCCGCTGCTCCAT

#### 2.3.4 Colorimetric assay

Following the removal of the medium, the cells were collected, and the extraction solution was added in accordance with the manufacturer’s instructions for the Copper Assay Kit (Elabscience, Cat# E-BC-K775-M). The supernatant was collected, and a portion was used for quantifying the protein. The copper concentration was calculated based on the optical density (OD) and protein concentration at a wavelength of 580 nm.

#### 2.3.5 Cell proliferation assay

The extracted CD8^+^ T cell suspension containing FBS was used to adjust the cell concentration, and CD8^+^ T cells were added to a 96-well plate with a circle of PBS to prevent evaporation. The wells were then incubated at 37 °C for 0, 24, 48, 72, and 96 h. Subsequently, 10 μL of MTS reagent (Saint-Bio Biotechnology, ST1009) was added and incubated at 37 °C for 2 h. OD was measured at 490 nm using an enzyme meter, and a curve was plotted.

#### 2.3.6 Transwell migration and invasion assay

For the migration experiments, the CD8^+^ T cell suspension was adjusted to the desired concentration. The lower chamber of the Transwell plates was filled with RPMI 1640 medium containing FBS, and the upper chamber was filled with a serum-free cell suspension. The cells were incubated at 37 °C and 5% CO_2_ for 24 h. After washing the membranes three times with PBS, they were fixed in 4% paraformaldehyde (Biomiky, MK014A) in the upper chamber. After washing with PBS, the membranes were stained with 10% crystal violet (Beyotime, C0121) in the upper chamber. The membranes were washed with PBS. Neutral resin sealing and microscopic examination were performed. For the invasion experiment, a matrix gel (Corning, 356234) was added to the upper chamber, and the remaining chambers were subjected to migration experiments.

#### 2.3.7 Western blotting

RIPA lysis solution (Beyotime, P0013B) containing protease and phosphatase inhibitors (Beyotime, P1050) was used to lyse the cells, and protein was quantified using the Bradford assay (Beyotime, P0006C). The samples were boiled and denatured by adding sodium dodecyl sulfate (SDS) loading buffer. Subsequently, electrophoresis, membrane transfer, blocking, washing with TBST, overnight incubation with the primary antibody, washing with TBST, incubation with the secondary antibody, washing with TBST, and ECL development (ZENBIO,17046) were performed. The membrane was developed after washing with TBST and processed using the ImageJ software. DLST, DLAT, and HSP70 were detected in CD8^+^ T cells; FN1 was detected in HSF cells; and α-SMA and CD31 were detected in HUVEC.

DLST(Human,P36957,Cell Signaling)DLAT(Human,AB172617,Abcam), HSP70(Human,10995-1-AP,Proteintech),GLS(Human,YN4051,Immunoway),Fibronectin 1(Human,YT1733,Immunoway),α-SMA(Human,YM8040,Immunoway),CD31(Human,YM8079,Immunowa-y),β-actin(Human,200068-8F10,ZENBIO),GAPDH(Human and Mouse,R380626,ZENBIO),Goat Anti-Rabbit IgG H&L(Human,511203,ZENBIO),Goat Anti-Mouse IgG H&L(Human,511103,ZENBIO),CD31(Mouse,ab222783,Abcam),α-SMA(Mouse,19245,CST),COL1A1(Mouse,BS60771,bioworld),Fibronectin 1(Mouse,MB63314,bioworld);β-actin(Mouse,380626,Zs-BIO);Goat anti-mouse IgG(Mouse,ZB-2305,Zs-BIO);Goat anti-rabbit IgG(Mouse,ZB-2301,Zs-BIO).

#### 2.3.8 ELISA

When the cells reached 80–90% confluence in the culture dish, they were cultured for 48–72 h and centrifuged to collect the culture supernatant. According to the manufacturer’s instructions (Jymbio Biotechnology, JYM0140Hu, JYM0151Hu, JYM0110Hu, and JYM0235Hu), OD was measured at 450 nm. Finally, the expression levels of IL-6, IL-13, CXCL3, and TNF-α were measured.

#### 2.3.9 Cell transfection

The concentration of Jurkat T cells was adjusted using Opti-MEM (ThermoFisher, 31985070), GLS-overexpressing virus was added for transfection (Feng Hui Biotechnology, China), and the culture was expanded by changing the medium after 24 h.

#### 2.3.10 Dual-luciferase reporter gene assay

Constructed wild-type (WT) and mutant-type (MUT) *GLS* and *TTLL7-IT1* (Feng Hui Biotechnology, China) were inoculated into 6-well plates with 293T cells. WT and MUT *GLS* and *TTLL7-IT1*, empty load, *Hsa-miR-29c-3p* mimics, and NC were mixed with Lipo 2000 (ThermoFisher,11668019), incubated at room temperature, transfected into 293T cells, and detected using a dual-luciferase detection kit (Beyotime Biotechnology, RG029S). Using sea cucumber luciferase as an internal reference, the RLU values from the firefly luciferase assay were divided by those from the sea cucumber luciferase assay and normalized. The groups were *TTLL7-IT1* WT+*hsa-miR-29c-3p* mimic, *TTLL7-IT1* WT+*hsa-miR-29c-3p* NC; *TTLL7-IT1* MUT+*hsa-miR-29c-3p* mimic, *TTLL7-IT1* MUT+*hsa-miR-29c-3p* NC; psicheck2+mimic, psicheck2+NC; *GLS* WT+*hsa-miR-29c-3p* mimic, *GLS* WT+*hsa-miR-29c-3p* NC; *GLS* MUT+*hsa-miR-29c-3p* mimic, *GLS* MUT+ *hsa-miR-29c-3p* NC; and psicheck2+*hsa-miR-29c-3p* mimic, psicheck2+*Hsa-miR-29c-3p* NC.

#### 2.3.11 Co-culture

CD8^+^ T cells from the lcSSc and lcSSc+TTM groups were placed separately in the upper layers of the two chambers, and HSF or HUVEC were added to the lower layer. After co-culturing for 24 h, the small chamber was removed, and culture was continued for HSF or HUVEC in the lower chamber.

### 2.4 Animal experiments

#### 2.4.1 Hematoxylin and eosin staining

Following treatment of skin sections with xylene and 100%–95%–80% ethanol, they were washed with alcohol, cleaned, stained with hematoxylin, washed with water, differentiated in a 1% hydrochloric acid alcohol solution, rinsed with water, blue-stained in a dilute lithium carbonate solution, washed with water, dehydrated using 80% ethanol, stained with eosin solution, toned using 95% ethanol I and II, dehydrated using anhydrous ethanol I and II, cleared in xylene I and II, and mounted for microscopic examination. Dermal thickness measurements were performed using the ImageJ software.

#### 2.4.2 Masson’s trichrome staining

After dewaxing the skin tissue sections in water, they were stained with Weigert’s iron hematoxylin. After differentiation with an acidic ethanol solution, the cells were rinsed with water, counterstained with Masson’s trichrome stain for 3–5 min, and rinsed with water. Subsequent steps included staining with Alcian blue, washing with a weak acid, washing with phosphomolybdic acid solution, another weak acid wash, direct staining in aniline blue solution, washing with a weak acid working solution, rapid dehydration with 95% ethanol, three rounds of dehydration with anhydrous ethanol, three rounds of clearing with xylene, and final mounting with neutral collodion for microscopic examination. The ImageJ software was used to measure the collagen fibers.

#### 2.4.3 Immunofluorescence

The skin was fixed in formalin, sectioned, incubated with primary and secondary antibodies, and examined microscopically. DAPI stained blue, CD31 emitted green light, and α-SMA emitted red light. The ImageJ software was used to analyze the percentage of co-localized areas in the images. (CD31, Abcam, Ab222783; α-SMA, CST, 19245t; Cy3-labeled goat anti-rabbit IgG (H^+^L), Beyotime, A0516; Goat Anti-Rabbit IgG (H^+^L) FITC, Affinity, S0008).

#### 2.4.4 RT-qPCR

*FN1, α-SMA, COL1A1*, and *GLS* expression in mouse skin was detected using RT-qPCR. Specific details are provided in Section 2.3.3.

#### 2.4.5 Western blotting

α-SMA, FN1, COL1A1, and GLS were detected in mice skin using western blotting. Specific details are provided in Section 2.3.7.

### 2.5 Data processing and statistical analysis

SPSS 23.0 was used for data analysis. The GraphPad Prism 10.1.2 software was used to generate the graphs. Mean ± standard deviation (for continuous data meeting normal distribution) and median + interquartile range (for ordinal data and continuous non-normally distributed data) were used for statistical description. Pearson correlation (for continuous data meeting normal distribution) and Spearman correlation (for ordinal data and non-normally distributed continuous data) were employed for correlation analysis. Independent samples t-test (normally distributed data with equal variances), Welch’s t-test (normally distributed data with unequal variances), and Mann-Whitney U test (continuous data not normally distributed) were used to compare two groups of data. One-way ANOVA (normal distribution, equal variances), Welch’s ANOVA (continuous data, normal distribution but unequal variances), and Kruskal-Wallis H test (continuous but non-normally distributed data, ordinal data) were used for testing among three groups of data. Statistical significance was set at *p <* 0.05, where *p < 0.05, **p < 0.01, ***p < 0.001, ****p < 0.0001.

### 2.6 Study approval

Written informed consent was obtained from all participants after receiving ethical approval from the Biomedical Ethics Committee of Anhui Medical University (code: 81250273). All animal experiments were conducted according to protocols approved by the Laboratory Animal Ethics Committee of Anhui Medical University (code: LLSC20250925).

### 2.7 Data availability

RNA-Seq data were deposited in the NBCI Gene Expression Omnibus Database (GEO: GSE308096). The values for all data points in the graphs are reported in the Supporting Data Values. All other data are available from the corresponding author upon reasonable request.

## 3 Results

### 3.1 Construction and validation of a cuproptosis-related ceRNA network in lcSSc

**Figure 1:**
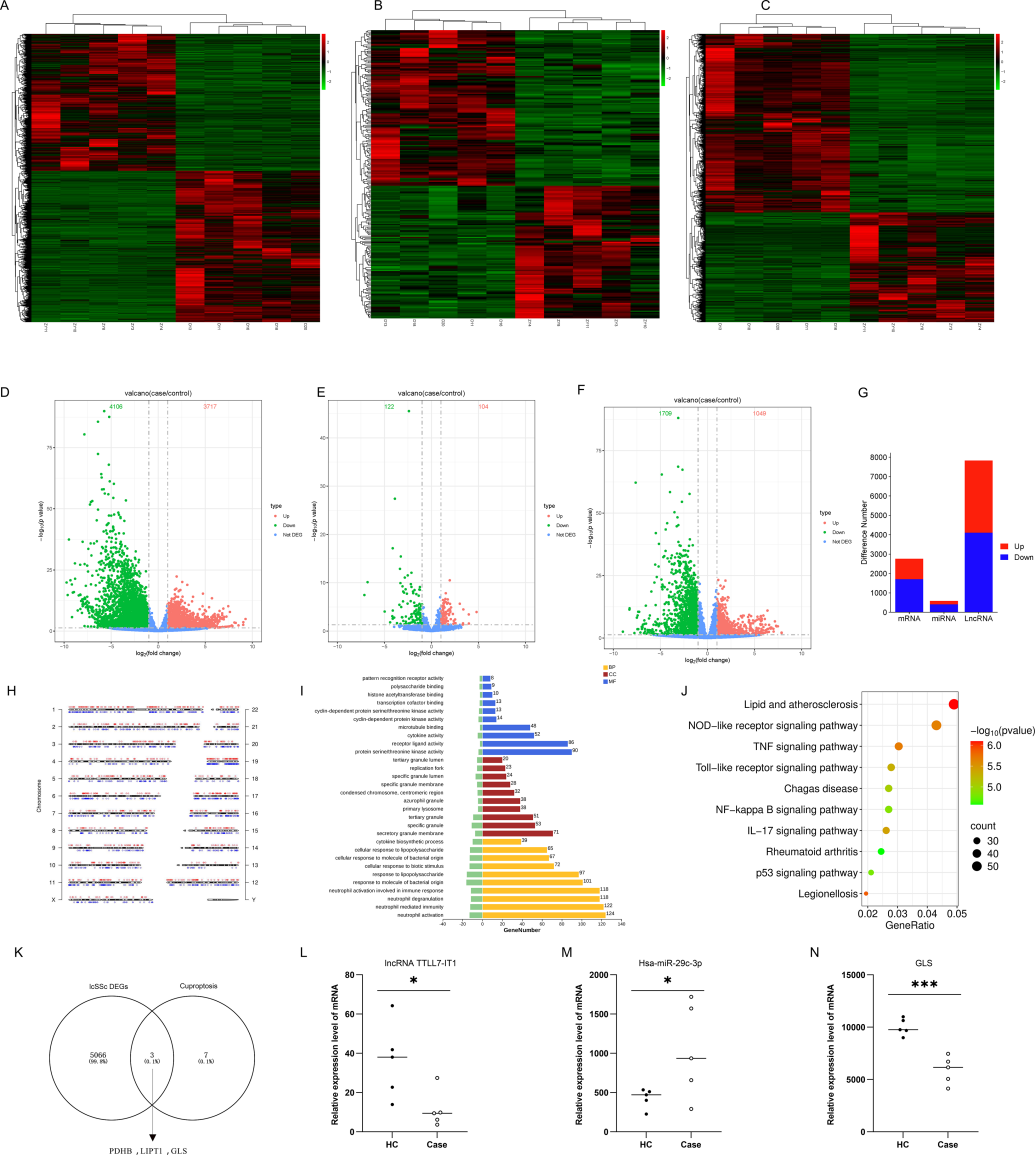
Preliminary findings of TTLL7-IT1/hsa-miR-29c-3p/GLS. **(A–F)** Heatmaps and volcano plots showing the differential distribution of mRNAs, miRNAs, and lncRNAs (n=5); **(G)** Histogram of the differential expression of mRNAs, miRNAs, and lncRNAs (n=5); **(H)** Chromosomal distribution of differentially expressed mRNAs (n=5); **(I)** Gene Ontology (GO) enrichment analysis of differentially expressed mRNAs (n=5); **(J)** Analysis of Differentially Expressed mRNA-Associated Pathways Using the Kyoto Encyclopedia of Genes and Genomes (KEGG) (n=5); **(K)** Venn diagram of cuproptosis-related genes and differentially expressed mRNA (n=5); **(L–N)** Histograms of the relative expression of lncRNA TTLL7-IT1, hsa-miR-29c-3p, and GLS in healthy controls (HC) and Case based on transcriptome sequencing data (n=5).

Using transcriptomic data, we identified TTLL7-IT1/hsa-miR-29c-3p/GLS. Transcriptomic sequencing data provided a descriptive profile of the samples (**Figures 1A–J**); identified numerous lncRNAs, miRNAs, and mRNAs; and revealed multiple pathways potentially associated with lcSSc pathogenesis through enrichment analysis, based on the pathways that exert their effects through ceRNA network regulation (27, 28). Following the intersection of differentially expressed mRNAs from our sequencing data with known cuproptosis-related genes, the cuproptosis-related genes *PDHB*, *GLS*, and *LIPT1* were identified (**Figure 1K**). Subsequently, we examined their upstream regulators and preliminarily constructed a ceRNA network related to apoptosis: lncRNA TTLL7-IT1/Hsa-miR-29c-3p/GLS. The expression levels of these network components are shown in **Figures 1L–N**. Compared with healthy controls, expression of *TTLL7-IT1* and *GLS* was significantly downregulated in lcSSc, whereas expression of *hsa-miR-29c-3p* was significantly upregulated.

**Figure 2:**
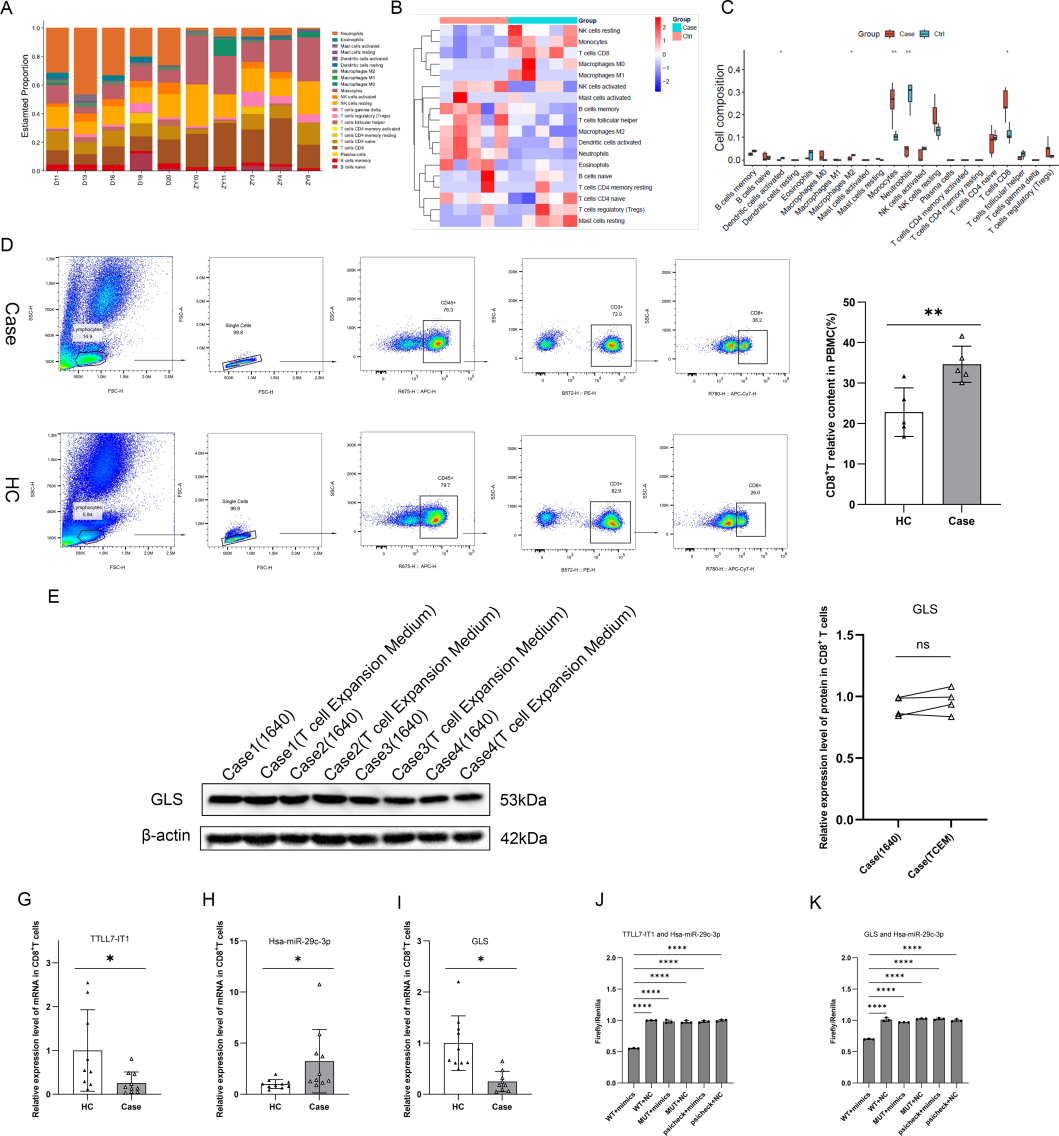
Validation of the TTLL7-IT1/hsa-miR-29c-3p/GLS network in lcSSc CD8^+^ T cells. **(A)** Bar plot of immune cell proportions by transcriptome data (n=5); **(B)** Heatmap of immune cell proportions by transcriptome data (n=5); **(C)** Box plot comparing immune cell abundance between Case and HC (n=5); **(D)** Flow cytometry analysis of the relative content of CD8^+^ T cells in the peripheral blood of Case and HC (n=5); **(E, F)** Effects of RPMI 1640 medium and T cell Expansion Medium (TCEM) on GLS expression during the activation of cultured CD8^+^ T cells (n=4); **(G–I)** Histograms of the relative expression of TTLL7-IT1 (Case: n=10, HC: n=9) hsa-miR-29c-3p (n=10), and GLS (Case: n=9, HC: n=10); **(J)** Histogram of dual-luciferase reporter assay results validating the putative binding site for hsa-miR-29c-3p on the lncRNA TTLL7-IT1; **(K)** Histogram of dual-luciferase reporter assay results validating the putative binding site for hsa-miR-29c-3p on the GLS. *p < 0.05; **p < 0.01; ***p < 0.001; ****p < 0.0001.

In addition, we used sequencing data to analyze the relative abundance of various immune cell types (**Figures 2A–C**), which revealed that CD8^+^ T cells comprised the largest proportion of infiltrating immune cells. Supporting this, a study reported elevated levels of CD8^+^ T cells in SSc (29), which aligns with the infiltration patterns. Therefore, we selected CD8^+^ T cells as the target population for this study. To validate the transcriptomic findings of the ceRNA network, we measured the CD8^+^ T cell content in the peripheral blood using flow cytometry. Examination of peripheral blood revealed a higher proportion of CD8^+^ T cells in patients with lcSSc than in HC (**Figure 2D**). We focused on CD8^+^ T cells for further analysis.

Given the important role of CD8^+^ T cells, we explored the existence of ceRNA networks in these cells. CD8^+^ T cells transform from a resting to an activated state when stimulated with CD3/28 and Interleukin-2 (IL-2). Concurrently, glutamine uptake and its catabolism were markedly upregulated. During this process, *GLS* expression, which is responsible for glutamine degradation, substantially increases (30). Given that *GLS* is a core gene in the predicted ceRNA network, we investigated whether the composition of the culture medium affected *GLS* expression by activating CD8^+^ T cells under the two medium conditions and assessed *GLS* expression in patient-derived CD8^+^ T cells. The results indicated no difference between the conditions, suggesting that the medium composition did not influence *GLS* changes during CD8^+^ T cell activation (**Figures 2E, F**).

RT-qPCR was performed to assess the expression levels of *hsa-miR-29c-3p*, *TTLL7-IT1*, and *GLS* genes. The results showed that both *GLS* (**Figure 2I**) and *lncRNA TTLL7-IT1* (**Figure 2G**) were significantly downregulated, whereas *hsa-miR-29c-3p* was upregulated in CD8^+^ T cells (**Figure 2H**). Based on the identified TTLL7-IT1/miR-29c-3p/GLS expression pattern, we sought to validate the direct molecular interactions in this axis. Using the StarBase 2.0 and BiBiServ databases, we predicted two binding sites for hsa-miR-29c-3p/TTLL7-IT1 and hsa-miR-29c-3p/GLS. To experimentally confirm these interactions, we constructed TTLL7-IT1 WT and MUT and GLS WT and MUT, which were grouped with hsa-miR-29c-3p mimics and NC. The cells were then simultaneously transfected with 293T cells. Reporter gene assays showed that the binding of *TTLL7-IT1* WT and *hsa-miR-29c-3p* mimics to WT-bound NC, MUT-bound mimics, and MUT-bound NC significantly reduced the luciferase activity. In addition, the binding of *GLS* WT to *hsa-miR-29c-3p* mimics was reduced compared with that in the other groups. The results indicated that *TTLL7-IT1* bound to and inhibited *hsa-miR-29c-3p*, in that *hsa-miR-29c-3p* bound to and negatively regulated *GLS* (**Figures 2J, K**). Finally, *TTLL7-IT1* inhibits *Hsa-miR-29c-3p*, and *Hsa-miR-29c-3p* in turn inhibits the expression of *GLS*, forming a connected network. This reciprocal expression pattern aligns with ceRNA regulatory principles and provides evidence for the functional existence of the TTLL7-IT1/hsa-miR-29c-3p/GLS axis in lcSSc CD8^+^ T cells.

In addition, we performed the modified Rodnan Skin Score(mRSS) on these patients and found that the skin involvement of those patients was characterized by sclerosis localized primarily to the fingers, hands, and forearms. The majority of individual lesions received a score of 1, while only a few were scored as 2. No lesion reached a score of 3. We then calculated the mRSS total score for each patient and analyzed its correlation with *GLS* expression in CD8^^+^^ T cells. The mRSS total score distribution was 4.5(2, 7.25). Spearman correlation analysis revealed a significant negative correlation between mRSS and *GLS* expression (*r_s_* = -0.975, *p* < 0.0001), indicating that lower *GLS* was associated with more severe skin fibrosis. Taking together, these findings suggested that the lncRNA TTLL7-IT1/Hsa-miR-29c-3p may contribute to lcSSc skin fibrosis through modulating *GLS* expression.

### 3.2 Cuproptosis results in the high proliferative, migratory, and invasive capacity of CD8^+^ T cells in patients with lcSSc, as well as the upward adjustment of ROS

**Figure 3:**
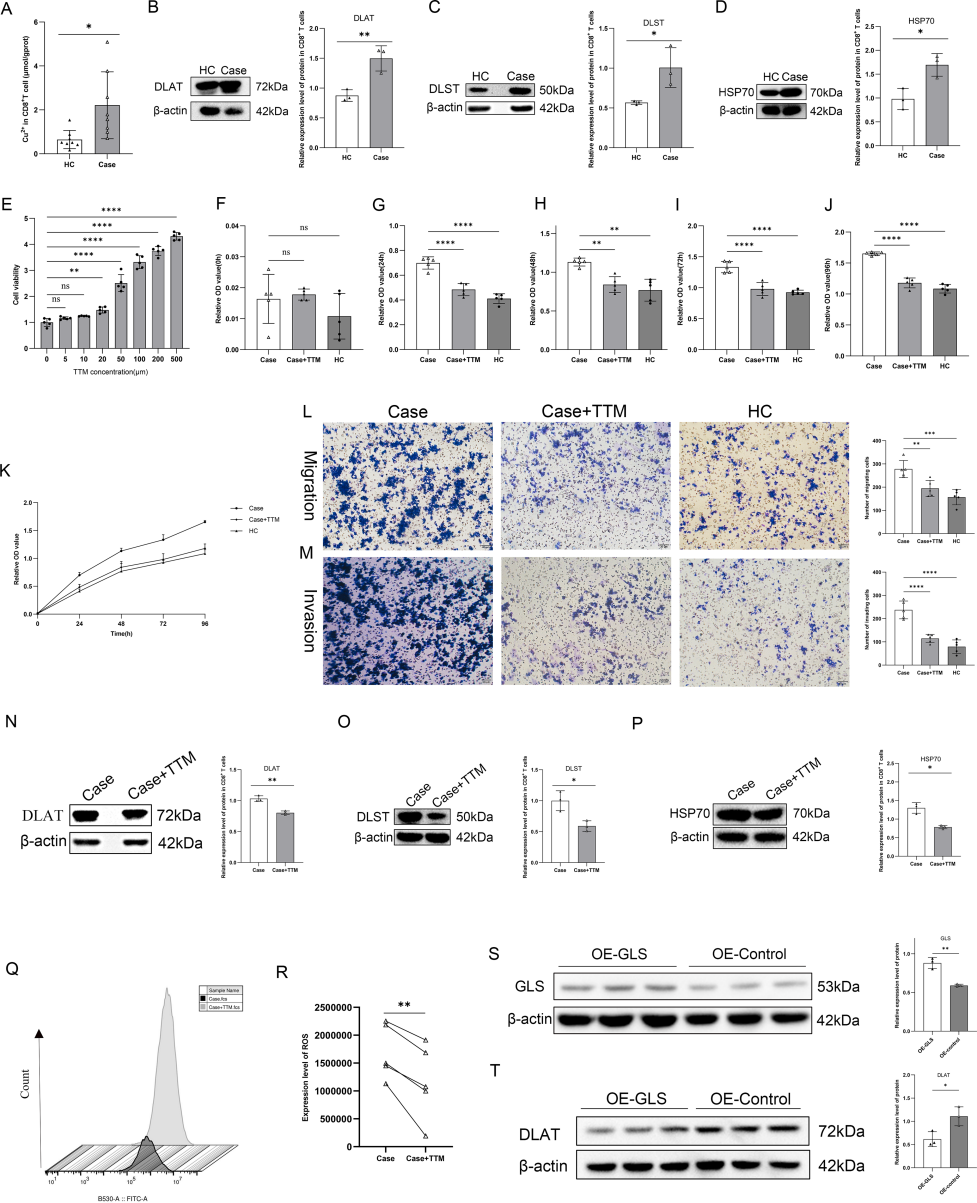
TTLL7-IT1/hsa-miR-29c-3p/GLS axis regulates CD8^+^ T cell function **(A)** Histogram of the difference in copper levels in CD8^+^ T cells between the Case and HC groups (n=8); **(B–D)** Bar charts of the relative protein expression in CD8^+^ T cells and corresponding differences in DLAT, DLST, and HSP70 in the HC and Case groups (n=3); **(E)** Histogram of the effect of TTM treatment concentration on Case CD8^+^ T cell activity; **(F–K)** Histogram of the proliferative capacity of CD8^+^ T cells at 0, 24, 48, 72, and 96 h among Case group, Case+TTM group, and HC group; **(L, M)** Microscopic images and statistical graphs of CD8^+^ T cell migration and invasion among Case group, Case+TTM group and HC group; **(N–P)** Bar charts of the relative protein expression levels in CD8^+^ T cells and corresponding differences in DLAT, DLST, and HSP70 in the Case and Case+TTM groups (n=3); **(Q, R)** Flow cytometry detection of relative expression abundance of ROS in CD8^+^ T cells in the Case group and Case+TTM group (n=5); **(S)** Expression levels and statistical histograms of GLS protein in OE-GLS and Control groups (n=3); **(T)** Expression levels and statistical histograms of the cuproptosis-related protein DLAT in OE-GLS and Control groups. *p < 0.05; **p < 0.01; ***p < 0.001; ****p < 0.0001.

To observe the connection between cuproptosis and CD8^+^ T cells in lcSSc, Cu^2+^ and cuproptosis-related proteins in CD8^+^ T cells were further examined, which revealed that the copper ion levels in CD8^+^ T cells of HC were significantly reduced compared to those in lcSSc CD8^+^ T cells (**Figure 3A**). Consistent with this, differential expression of cuproptosis-related proteins (DLAT, DLST, and HSP70) was observed between the HC and lcSSc groups. Given that these proteins are key markers of apoptosis, these findings preliminarily indicate that cuproptosis occurs in CD8^+^ T cells (**Figures 3B–D**).

As a preliminary assessment, CD8^+^ T cells were treated for 24 h with a range of TTM concentrations (TTM can bind copper as a chelating agent and inhibits apoptosis). Based on these preliminary results, 20 μM TTM effectively inhibited cuproptosis without excessive toxicity; therefore, we selected this concentration for subsequent experiments (**Figure 3E**). The proliferative capacity of the cells was assessed using the MTS assay at 0, 24, 48, 72, and 96 h. The results indicated that the proliferative capacity of CD8^+^ T cells from patients with lcSSc was higher than that of cells from HC and was higher than that of TTM-treated lcSSc cells after 24 h. This trend persisted for 96 h post-treatment. These findings suggest that suppression of the apoptotic pathway reduces the proliferative capacity of lcSSc CD8^+^ T cells. (**Figures 3F–K**). To evaluate the impact of TTM on the migration and invasion capabilities of CD8^+^ T cells from patients with lcSSc, we conducted Transwell assays (**Figures 3L, M**). The results indicated that the migration and invasion capacities of CD8^+^ T cells were higher in lcSSc than in TTM-treated patients and HC. Inhibition of cuproptosis led to a marked reduction in the proliferative, migratory, and invasive capacities of CD8^+^ T cells from patients with lcSSc, as shown by these collective findings. TTM treatment, aimed at rescuing copper-induced apoptosis, decreased DLAT, DLST, and HSP70 expression. These findings show that cuproptosis was present in the CD8^+^ T cells of patients with scleroderma (**Figures 3N–P**). In addition, ROS are key factors in the onset of lcSSc, and we hypothesized a link between ROS and lcSSc. Verification assays showed an increase in ROS levels in CD8^+^ T cells after cuproptosis (**Figures 3Q, R**). These results revealed that cuproptosis regulates CD8^+^ T cell function.

The confirmed occurrence of cuproptosis in lcSSc CD8^+^ T cells, and evidence from the literature that acute *in vitro* inhibition or knockout of GLS impairs the normal immune function of CD8^+^ T cells (30), revealing that low GLS expression affects CD8^+^ T cell function, prompted us to further explore the regulatory mechanisms of GLS in cuproptosis. We selected Jurkat T cells for GLS to study their effects on apoptosis because of their stable reproducibility, suitability for molecular interactions, and previous studies that used Jurkat T cells to replace primary T cells for mechanistic research (31). Overexpression of GLS (OE-GLS) in Jurkat T cells led to a decrease in DLAT protein levels (**Figures 3S, T**). This may represent a novel mechanism for the inhibition of apoptosis. Reduced DLAT may lead to a decrease in pyruvate dehydrogenase complex activity, preventing pyruvate from being effectively converted into acetyl-CoA, reducing the TCA cycle flux, mitochondrial ATP production, and ROS production, simultaneously increasing the stability of Fe-S cluster proteins, reducing protein toxicity and inhibiting apoptosis. TTLL7-IT1/hsa-miR-29c-3p mediates cuproptosis via GLS, affecting CD8^+^ T cell function.

### 3.3 The cuproptosis pathway affects the fibrosis, vascular lesions, and immune dysregulation of lcSSc through CD8^+^ T cells

**Figure 4:**
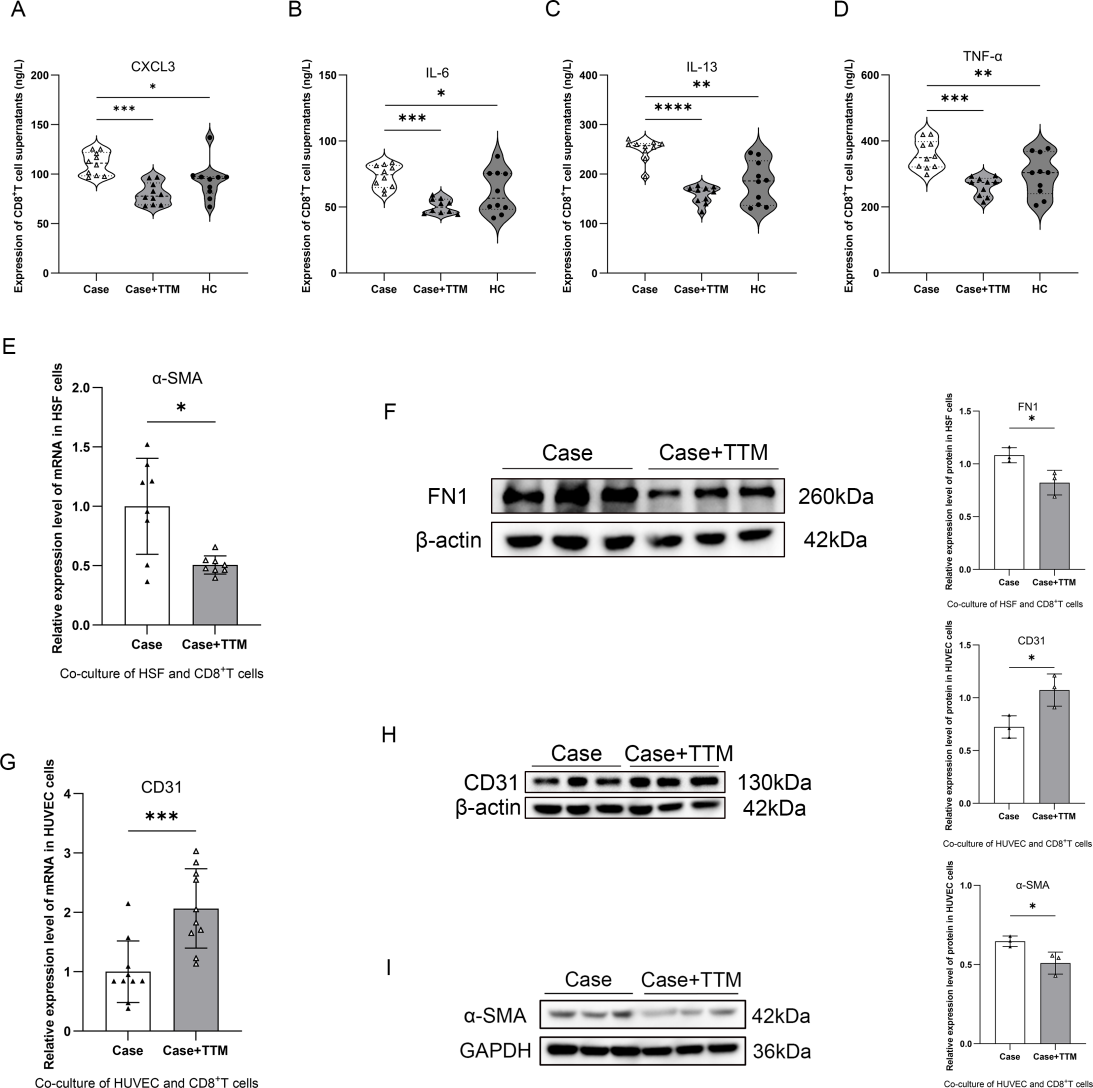
Cuproptosis affects lcSSc onset. **(A–D)** Violin plots showing CXCL3, IL-6, IL-13, and TNF-α expression in CD8^+^ T cells from HC, Case, and Case+TTM groups (n=10); **(E)** qPCR analysis of α-SMA mRNA in co-cultured CD8^+^ T cells and HSF from Case and Case+TTM groups (n=8); **(F)** Western blot analysis of FN1 protein in co-cultured CD8^+^ T cells and HSF from Case and Case+TTM groups (n=3); **(G)** qPCR analysis of CD31 mRNA in co-cultured CD8^+^ T cells and HUVEC from Case and Case+TTM groups (n=10); **(H)** Western blot analysis of CD31 protein in co-cultured CD8^+^ T cells and HUVEC from Case and Case+TTM groups (n=3); **(I)** Western blot analysis of α-SMA protein in co-cultured CD8^+^ T cells and HUVEC from Case and Case+TTM groups (n=3). *p < 0.05; **p < 0.01; ***p < 0.001; ****p < 0.0001.

Given the critical role of cytokine dysregulation in lcSSc pathogenesis, we quantified the cytokine levels secreted by CD8^+^ T cells across three groups: lcSSc, lcSSc+TTM, and HC (n=10). The results showed that IL-13 levels were higher in CD8^+^ T cells from untreated patients with lcSSc than in those from HC; however, following TTM treatment, Interleukin-13 (IL-13) levels were downregulated relative to those in the untreated lcSSc group. Similarly, we observed significant upregulation trends for Interleukin-6 (IL-6), C-X-C motif chemokine ligand 3 (CXCL3), and TNF-α within this context; conversely, treatment with TTM markedly reduced the secretion of IL-6, CXCL3, and TNF-α (**Figures 4A–D**) in CD8^+^ T cells derived from patients with lcSSc. Previously identified IL-13, IL-6, CXCL3, and TNF-α support our findings regarding their roles in SSc (32–35). Therefore, cuproptosis can exacerbate the onset of lcSSc by increasing the levels of IL-13, IL-6, CXCL3, and TNF-α.

As a key component of the immune system, CD8^+^ T cell involvement in fibrosis and endothelial pathological changes has been documented in several studies (36). However, studies exploring the direct effects of cuproptosis on fibrosis and angiogenesis in patients with lcSSc are scarce. To explore this relationship, we conducted co-culture experiments using Transwell systems, in which co-cultures were established between activated CD8^+^ T cells and two cell types: HUVEC and HSF. HUVEC or HSF were placed in the lower chamber, whereas activated CD8^+^ T cells occupied the upper chamber. Although the membranes of the chambers were used to separate CD8^+^ T cells and HSF or HUVEC in the co-culture, being part of the same culture system made it impossible to distinguish cell-specific secretions and quantify cytokine changes in the cell supernatant measured using ELISA. Following co-culture, we expanded HUVEC and HSF separately and assessed changes in fibrosis-related and angiogenesis-related markers at both the protein (via western blotting) and mRNA (via qPCR) levels.

After co-culturing with HSF cells of the two groups, the results revealed Fibronectin 1 (FN1) and α-smooth muscle actin (α-SMA) in HSF showed varying degrees of decrease in the lcSSc+TTM group compared to untreated lcSSc group (**Figures 4E, F**), which are key factors in fibrosis, indicating that cuproptosis in CD8^+^ T cells promote the secretion of fibrotic factors and may contribute to lcSSc pathogenesis. A significant increase in α-SMA gene expression was observed in the lcSSc group compared to that in the lcSSc+TTM group, indicating that cuproptosis may increase fibroblast activation, facilitating their transformation into myofibroblasts and contributing to tissue/organ sclerosis. Elevated FN1 levels in the lcSSc group indicated that FN1 may enhance extracellular matrix synthesis and collagen secretion, promoting fibrotic lesions in the skin and other organs. Consistent with this, cuproptosis-induced upregulation of FN1 further promotes the secretion of fibrotic materials, contributing to SSc progression (37). In contrast, the cell adhesion molecule CD31 is pivotal for angiogenesis and the preservation of vascular homeostasis. In HUVEC, CD31 expression was reduced in the lcSSc group compared to that in the TTM co-culture group (**Figures 4G, H**), indicating that cuproptosis in lcSSc may diminish cell adhesion and increase vascular permeability. As CD31 is involved in angiogenesis, inhibition of its secretion via the copper-induced necrotic pathway could reduce neovascularization (38, 39). α-SMA protein levels were significantly elevated in HUVEC from the lcSSc group compared to those from the lcSSc+TTM group after co-culture with HUVEC (**Figure 4I**). The cuproptosis-associated upregulation of α-SMA in HUVEC may enhance vascular migration and contraction, leading to vascular structural abnormalities and fibrosis (40, 41). These findings reveal that the apoptotic pathway affects inflammation, fibrosis, and angiogenesis in lcSSc via CD8^+^ T cells.

### 3.4 Blockade of cuproptosis ameliorates fibrosis and vascular lesions in the lcSSc mice model

**Figure 5:**
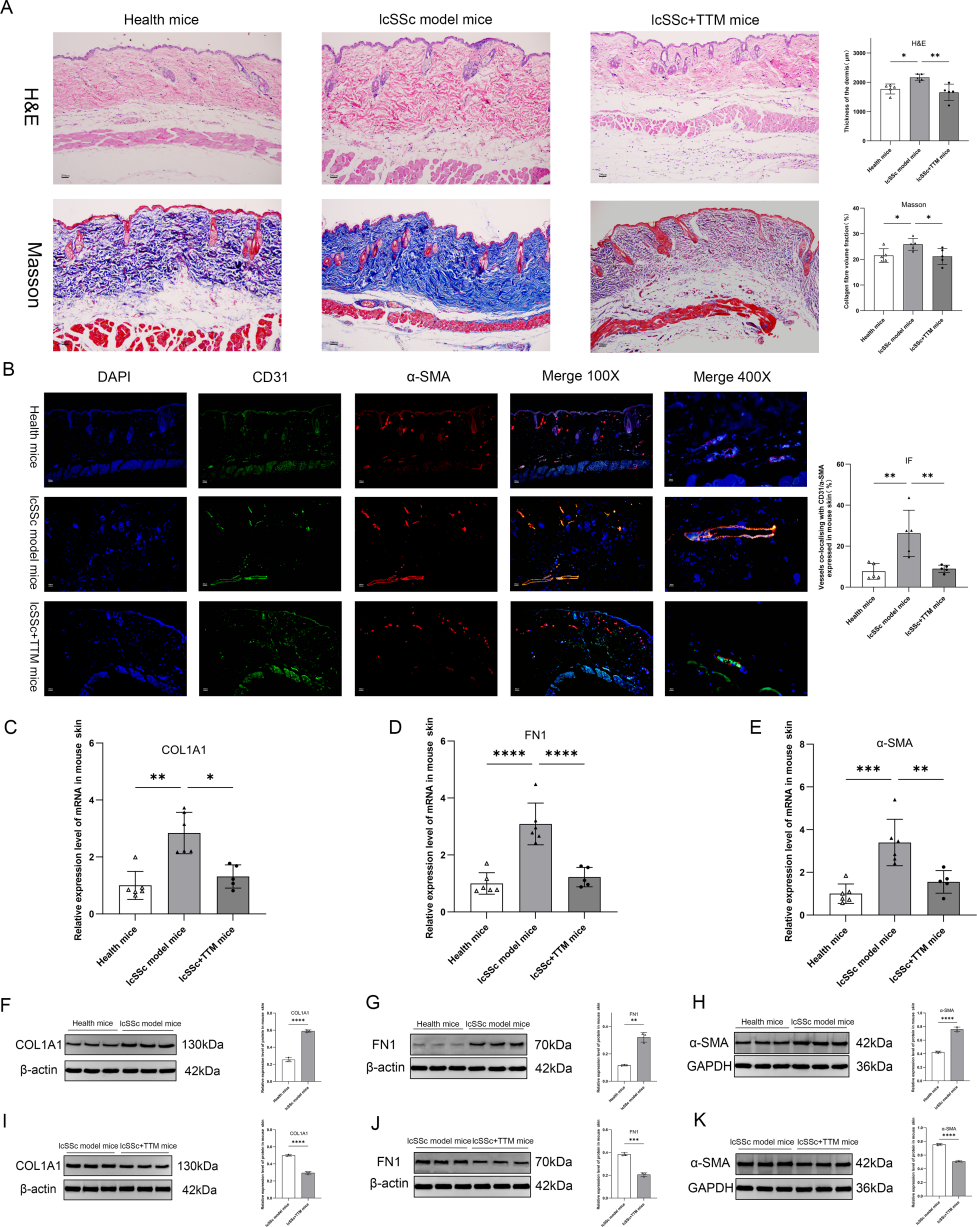
Validation of the lcSSc mouse fibrosis model and TTM-blocked cuproptosis alleviated fibrosis and vascular lesions. **(A)** H&E and Masson’s trichrome staining to assess skin dermis thickness and collagen fibers in healthy mice, lcSSc mice model, and lcSSc+TTM mice (n=5); **(B)** Immunofluorescence analysis of CD31/α-SMA co-localization percentage in the skin of health mice, lcSSc mice model, and lcSSc+TTM mice (CD31 is green fluorescence, α-SMA is red, and DAPI is blue) (n=5); **(C–E)** Analysis of COL1A1, FN1, and α-SMA gene expression using RT-qPCR in the skin of healthy mice (n=6), lcSSc-model mice (n=6), and lcSSc+TTM mice (n=5); **(F–H)** Western blot analysis of COL1A1, FN1, and α-SMA protein expression in skin from healthy mice and lcSSc-model mice (n=3); **(I–K)** Western blot analysis of COL1A1, FN1, and α-SMA protein expression in skin from lcSSc-model mice and lcSSc+TTM mice (n=3). *p < 0.05; **p < 0.01; ***p < 0.001; ****p < 0.0001.

Based on previous findings on cuproptosis, we performed additional *in vivo* experiments to determine its role. As the animal experiment was based on a bleomycin-induced skin fibrosis model, we validated the BLM-induced SSc mouse model. H&E staining of the skin tissues (**Figure 5A**) revealed that the dermis of mice in the lcSSc-model group was significantly thicker than healthy mice (without BLM injection). Masson staining to assess collagen fiber content (**Figure 5A**) revealed that compared to the health mice group, the volume fraction of collagen fibers in the lcSSc mice model increased significantly. Under normal circumstances, CD31 and α-SMA rarely co-localize in tissues. However, increased co-localization indicates endothelial–mesenchymal transition (EndoMT) in specific tissues. EndoMT leads to impaired endothelial cell function and detrimental changes in the vascular structure, resulting in vascular damage. Moreover, it promotes fibrosis in human cells (42, 43). Therefore, immunofluorescence was used to detect the co-localization of CD31 and α-SMA in skin tissues from both health and lcSSc-model mice. The co-localization effect of CD31/α-SMA in the skin of the lcSSc group was significant, and the percentage of positive co-localization increased (**Figure 5B**). In addition, assessment of fibrosis marker expression showed that *α-SMA, FN1*, and *COL1A1* levels in the skin of the lcSSc model were higher than those in the health mouse group (**Figures 5C–E, F, G**). These results confirmed the successful establishment of an lcSSc skin fibrosis mouse model.

We sought to determine the role of cuproptosis inhibition in the development of skin fibrosis. H&E and Masson staining (**Figure 5A**) revealed that the dermis of TTM-treated mice was significantly thinner than that of the lcSSc mice. Assessment of collagen fibers indicated that the administration of the cuproptosis inhibitor TTM significantly attenuated dermal collagen deposition in mice with experimental lcSSc. After TTM treatment, *α-SMA, COL1A1*, and *FN1* levels were significantly decreased (**Figures 5C–E, I–K**).

In addition, we assessed the co-localization of CD31/α-SMA in lcSSc-model mice using TTM and immunofluorescence (**Figure 5B**). Compared to lcSSc-model mice, the co-localization positive percentage of CD31/α-SMA in dermal vessels of lcSSc+TTM mice was decreased. Decreased CD31/α-SMA co-localization indicates the attenuation of EndoMT, a process that compromises angiogenesis. These results indicate that TTM improves vascular function and promotes angiogenesis in patients with lcSSc. Inhibition of apoptosis alleviates fibrosis and vascular lesions, indicating that cuproptosis exacerbates these conditions.

### 3.5 Overexpression of GLS attenuates fibrosis and vascular lesions in lcSSc mice

**Figure 6:**
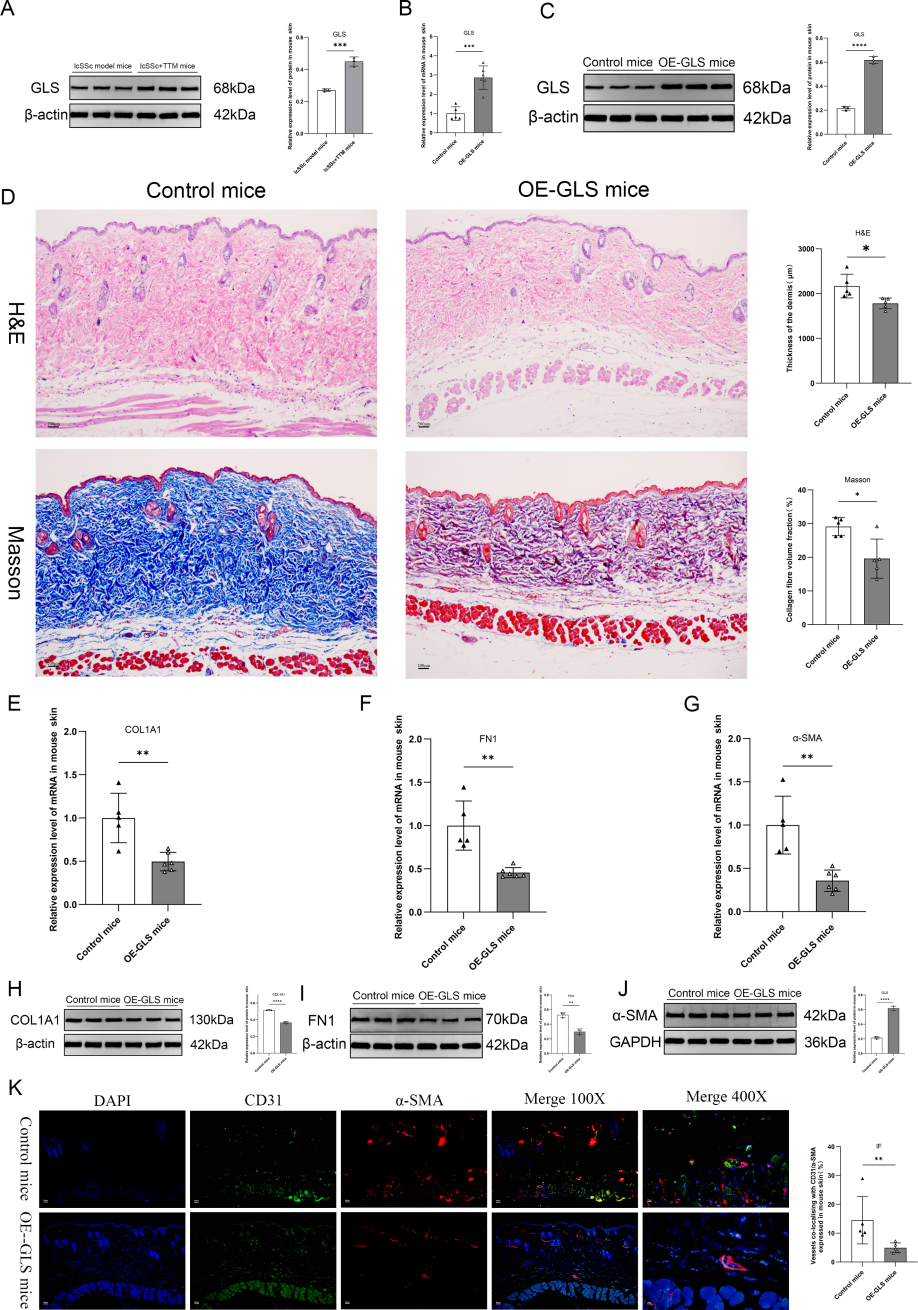
Overexpression of the cuproptosis-related gene GLS alleviates fibrosis and vascular lesions in lcSSc-model mice. **(A)** Analysis of GLS protein expression using western blotting in the skin of lcSSc-model mice and lcSSc+TTM mice (n=3); **(B)** RT-qPCR analysis of the expression of the GLS gene in the skin of control (n=5) and OE-GLS mice (n=6); **(C)** Western blot analysis of GLS protein expression in the skin of control and OE-GLS mice (n=3); **(D)** H&E and Masson staining of the dermal layer thickness and collagen fibers in the skin of control and OE-GLS mice (n=5); **(E–G)** RT-qPCR analysis of the expression of COL1A1, FN1, and α-SMA genes in the skin of control (n=5) and OE-GLS mice (n=6); **(H–J)** Western blot analysis of COL1A1, FN1, and α-SMA proteins in the skin of control and OE-GLS mice (n=3); **(K)** Immunofluorescence analysis of CD31/α-SMA co-localization percentage in the skin of control and OE-GLS mice (n=5).*p < 0.05; **p < 0.01; ***p < 0.001; ****p < 0.0001.

GLS expression showed a rebound trend in the lcSSc+TTM group compared to the lcSSc model after cuproptosis was blocked (**Figure 6A**), indicating that GLS may be a key cuproptosis-related gene that affects lcSSc. Therefore, to further characterize the effect of *GLS* on lcSSc, we overexpressed *GLS* in the lcSSc mouse model (**Figures 6B, C**). Compared with the control group (injected with overexpressed blank virus), the dermis of *GLS*-overexpressing mice was thinner, and the volume fraction of collagen fibers was significantly reduced (**Figure 6D**). *GLS* overexpression led to a significant downregulation of *α-SMA, COL1A1*, and *FN1* in the skin compared to the controls (**Figures 6E–G, H–J**), suggesting that *GLS* overexpression might attenuate the increased levels of lcSSc fibrosis. We explored the effect of *GLS* on vascular lesions. Immunofluorescence (**Figure 6K**) showed that, compared with the control group, the percentage of dermal vessels positive for CD31/α-SMA co-localization decreased in the *GLS*-overexpression group, indicating that *GLS* overexpression significantly alleviated vascular lesion formation. Low *GLS* expression may be involved in the vascular abnormalities associated with lcSSc. Therefore, *GLS* overexpression may alleviate fibrosis and vascular lesion formation.

## 4 Discussion

In this study, we detected TTLL7-IT1/hsa-miR-29c-3p/GLS expression in CD8^+^ T cells from patients with lcSSc. Low expression of *TTLL7-IT1* negatively regulates *Hsa-miR-29c-3p*, leading to its upregulation; in turn, the upregulation of *Hsa-miR-29c-3p* negatively regulates *GLS*, resulting in its downregulation, thereby forming a ceRNA network. Subsequently, the ceRNA network with low *GLS* expression triggers high *DLAT* expression through negative feedback, thereby exacerbating the copper-induced cell death process. This ultimately leads to increased fibrosis and vascular lesions, contributing to the progression of lcSSc.

We identified a key intronic transcript, *TTLL7-IT1*, which serves as an important molecule for lcSSc prevention and treatment. Although its precise role remains unclear, the main function of the TTLL (Tubulin Tyrosine Ligase-Like) family is to catalyze the post-translational modification of tubulin (44). As a component of the TTLL family, TTLL7, a β-microtubule protein polypeptidylglycosylase with the highest transcriptional level among TTLL, has been implicated in T cell activation and inflammatory responses (45). Although no studies have reported intron transcription in TTLL7, research has been conducted on the intron transcription of other genes. *lncRNA CPS1-IT1* interacts with HSP90 to regulate HIF-1α activity, promoting epithelial-mesenchymal transition (EMT). Similarly, *lncRNA FTO-IT1*, which is involved in the TCA cycle, also promotes glycolysis in hepatocellular carcinoma cells. In addition, *SPRY4-IT1* enhances the expression of genes related to EMT in cancer (46–48). These findings indicate that lncRNAs transcribed from introns regulate processes central to fibrosis, supporting the hypothesis that *TTLL7-IT1* influences fibrosis and angiogenesis in HSCs. Our study complements existing knowledge on the role of *TTLL7-IT1* in lcSSc and provides a basis for future research.

The other ceRNA network components, *hsa-miR-29c-3p* and *GLS*, are also critical for cuproptosis. In this study, *hsa-miR-29c-3p* was observed to target *GLS* through a ceRNA network, implicating it in the regulation of cuproptosis and lcSSc pathogenesis. Previously, *hsa-miR-29c-3p* has been used as a serum autoantibody biomarker for SSc (11). In contrast to our study, *miR-29c-3p* exerts an antagonistic effect in breast cancer, with its high expression inhibiting the regulation of copper ion transport (49). This may be due to differences in disease type; however, it highlights the crucial role of *hsa-miR-29c-3p* in cuproptosis. In addition, our study underscores the pivotal role of *GLS*, a gene associated with cuproptosis. *GLS* facilitates the production of glutamate from glutamine and influences ROS production and energy metabolism, with broad consequences (50). In addition, low *GLS* levels in alveolar macrophages have been linked to copper transport dysfunction, contributing to metabolic dysfunction in chronic obstructive pulmonary disease and supporting the role of *GLS* in copper homeostasis. Consistent with our findings, this indicates that low *GLS* expression may lead to cuproptosis.

lcSSc diagnosis relies primarily on the presence of inflammation, fibrosis, and vascular lesions. Based on diagnostic findings, traditional treatment approaches primarily target established mid-to-late stage disease, offering only symptom relief and progression delay, but with limited efficacy in reversing the condition (51–53). In patients in the middle-to-late stages, these pathological changes usually become apparent only after the organs or tissues have undergone significant fibrosis or lesions, and treatment cannot achieve the desired results. Therefore, following diagnosis, timely intervention with copper chelators or components of the ceRNA network may serve as a potential therapeutic approach to prevent copper-induced apoptosis. This could prevent immune dysregulation, release of profibrotic factors, and formation of vascular lesions triggered by this pathway. Dysregulation of molecular networks within CD8^+^ T cells can drive alterations in the immune microenvironment through cuproptosis, which may precede the onset of clinical symptoms. Therefore, monitoring the expression profiles of *TTLL7-IT1* or assessing cuproptosis in CD8^+^ T cells may enable early prediction of lcSSc. In addition, before the onset of pronounced symptoms, consideration should be given to elevating *TTLL7-IT1* levels in high-risk populations to modulate cutaneous cuproptosis and prevent symptom development.

Our research provides crucial scientific evidence for the prevention and treatment of lcSSc and offers novel approaches for early interventions. However, translating these findings into clinical practice requires further validation through additional research.

This study has a few limitations. First, we only included cases of lcSSc with no other subtypes. Second, the cases primarily originated from hospitals in Anhui Province, which may have introduced representativeness and regional bias. Third, following the extraction of CD8^+^ T cells, subsequent experiments were conducted without further characterizing the functional subsets. Fourth, additional cuproptosis-related factors have not yet been modulated, nor have the levels of *TTLL7-IT1* or *miR-29c-3p* been altered, to observe subsequent changes in downstream molecules and functions. We will conduct further research based on this study to address its limitations and rigorously investigate the role of CD8^+^ T cells in lcSSc pathogenesis.

## Data Availability

The datasets presented in this study can be found in online repositories. The names of the repository/repositories and accession number(s) can be found below: https://www.ncbi.nlm.nih.gov/, GSE308096.
